# Using fractal self‐similarity to increase precision of shrub biomass estimates

**DOI:** 10.1002/ece3.7393

**Published:** 2021-03-18

**Authors:** Roman J. Dial, Bethany Schulz, Eric Lewis‐Clark, Kaili Martin, Hans‐Erik Andersen

**Affiliations:** ^1^ Institute of Culture and Environment Alaska Pacific University Anchorage AK USA; ^2^ USDA Forest Service Pacific Northwest Research Station Anchorage AK USA; ^3^ USDA Forest Service Pacific Northwest Research Station University of Washington Seattle WA USA

**Keywords:** allometry, biomass, error propagation, fractals, self‐similarity, shrubs

## Abstract

We show that aerial tips are self‐similar fractals of whole shrubs and present a field method that applies this fact to improves accuracy and precision of biomass estimates of tall‐shrubs, defined here as those with diameter at root collar (DRC) ≥ 2.5 cm. Power function allometry of biomass to stem diameter generates a disproportionate prediction error that increases rapidly with diameter. Thus, biomass should be modeled as a single measure of stem diameter only if stem diameter is less than a threshold *D_max_*. When stem diameter exceeds *D_max_*, then the stem internode should be treated as a conic frustrum requiring two additional measures: a second, node‐adjacent diameter and a length. If the second diameter is less than *D_max_*, then the power function allometry can be applied to the aerial tip; otherwise an additional internode is measured. This “two‐component” allometry—internodes as frustra and aerial tips as shrubs—can reduce estimated biomass error propagated to the plot‐level by as much as 50% or more where very large shrubs are present *D_max_* is any diameter such that the ratio of single‐component to two‐component uncertainty exceeds the ratio of two‐component to single‐component measurement time. Guidelines for estimating *D_max_* based on pilot field data are provided. Tall shrubs are increasing in abundance and distribution across Arctic, alpine, boreal, and dryland ecosystems. Estimating their biomass is important for both ecological studies and carbon accounting. Reducing field‐sample prediction error increases precision in multi‐stage modeling because additional measures efficiently improve plot‐level biomass precision, reducing uncertainty for shrub biomass estimates.

## Graphical abstract

Shrub biomass is an increasingly important measure in changing ecosystems. We present a simple method of reducing errors in its estimation.

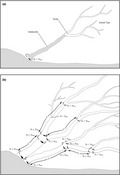

## 1 INTRODUCTION

**FIGURE 1:**
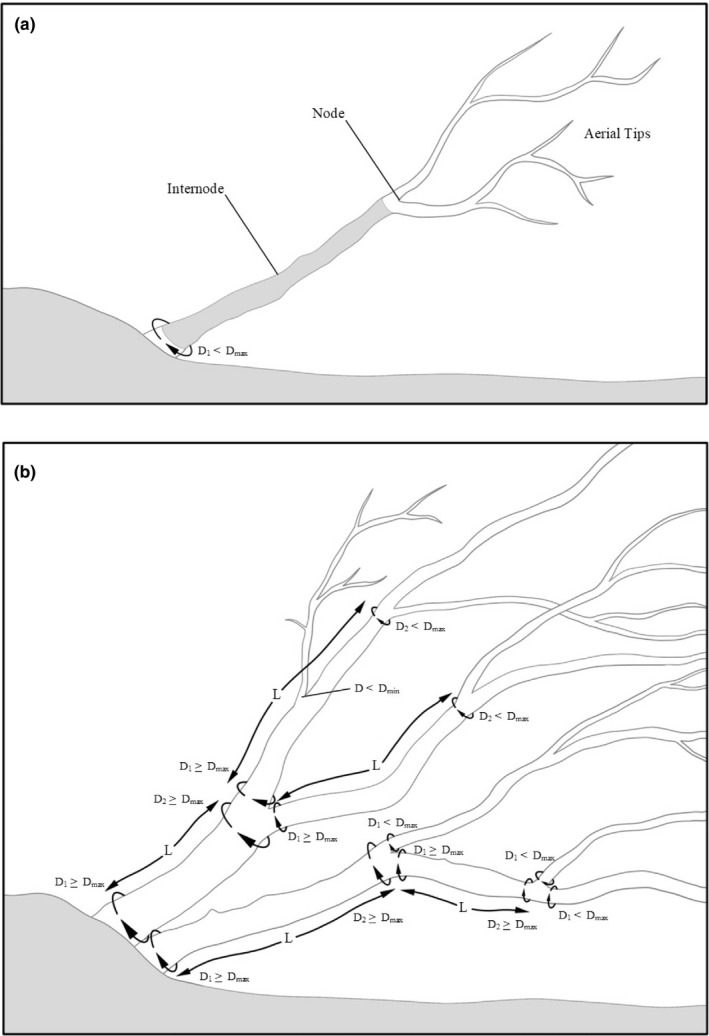
Two‐component sampling scheme that increases precision and accuracy in shrub biomass allometry. (a) Shrubs sampled as internodes and aerial tips. (b) Aerial tips are defined as those with supra‐nodal diameter less than a threshold (*D_max_*), determined as a breakpoint for uncertainty in power function allometry. Illustration by Julia Ditto

Ever since Julian Huxley introduced the concept of allometry in 1932, biologists have related one physical trait of an organism to another using power functions. Foresters, for example, quantify forest stand wood volume using diameter at breast height (DBH) and ecologists increasingly apply allometry to calculate shrub biomass (Berner et al., 2015; Chojnacky & Milton, 2008; Conti et al., 2019) when estimating landscape biomass (Alonzo et al., 2020; Berner et al., 2018). Species‐specific allometry (Paul et al., 2015) is readily available for trees (Woodall et al., 2011); however, fewer species‐specific equations are available for shrubs (Chojnacky & Milton, 2008). Because increasing populations of tall shrubs (Figure 1) grow in remote regions, quantifying their abundance relies on remote sensing informed by application of allometry to field data, thus leaving abundance estimates sensitive to propagated errors.

Expressed as a power function, biomass‐diameter allometry is theoretically reasonable if a relative growth increment in a volume‐related measurement, such as wet field‐mass *M*, is proportional to a relative growth increment in a linear‐related measurement, like diameter *D*. The relationship ΔM/M=bΔD/D implies that a sample of wet field‐mass for individual‐*i* follows ln(*M_i_*) = *b* ln(*D_i_*) + ln(*a*) + ε_i_, where ln(*a*) is the constant of integration, and 2 < *b* < 3 captures the self‐similar, fractal geometry of woody vegetation.

**FIGURE 2:**
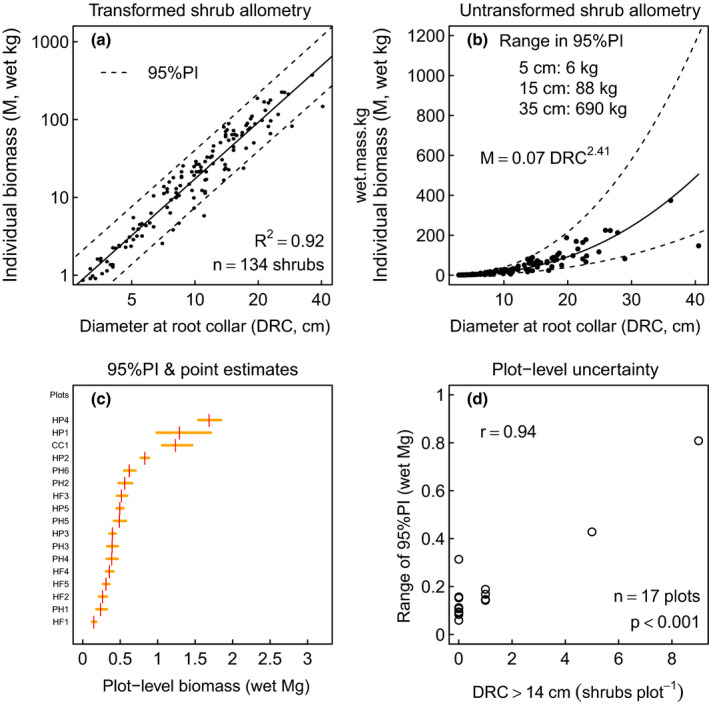
Uncertainty propagated from individual to the sample‐plot using whole‐shrub (single‐component) DRC‐based allometry. (a) Log‐log based allometry of shrub wet field‐mass (*M*) on DRC (*D*) as linear model ln(*M_i_*) *= b* ln(*D_i_*) + ln(*a*) + ε_i_. Dashed lines give 95% prediction interval (95%PI). (b) Unlogged data with allometry and 95%PI, highlighting expanding uncertainty (dashed lines). (c) Uncertainty of individual shrubs propagated to sample‐plot uncertainty for 17 circular 169 m^2^ plots (identified as three letter codes, where first two letters signify study site) in southcentral Alaska. “Plot‐level uncertainty” defined as range of 95%PI. Horizontal line segments connect upper (0.975) and lower (0.025) PI calculated as quantiles from Monte Carlo sampling; they are asymmetric about point estimates (vertical lines) due to power function allometry. (d) Plot‐level uncertainty plotted against the per sample‐plot count of large shrubs (*DRC* > 14 cm). Large shrubs increase uncertainty in plot‐level estimates of biomass

Because biomass is strictly positive, its mean and variance increase together. This nonindependence of point estimate (e.g., mean) and uncertainty (e.g., standard error) implies that fitting power functions with log‐log plots can help control for heteroscedastic residuals (Kerkhof & Enquist, 2009; Xiao et al., 2011), as can **nls()** with weighted regression (e.g., Berner et al., 2015). In practice, the biomass of a large shrub predicted from its allometry could have orders of magnitude more uncertainty than a smaller shrub (Figure 2b). This mathematically expanding uncertainty reflects the reality of plant ecology and individual plant life‐history (Kerkhof & Enquist, 2009). The problem is to accurately capture variability in plot‐level shrub biomass while minimizing uncertainty in individual‐plant biomass estimates subject to sampling time. The field‐sampling method presented here and shown in Figure 1 offers several advantages. It is independent of curve‐fitting approach, reduces uncertainty, and improves accuracy by performing measurements that take advantage of the fractal nature of shrub architecture.

Applications of allometry that scale‐up from the individual to the plot‐level and beyond require methods that reduce propagated error. As an example, whole‐shrub biomass‐DRC (“one‐component”) allometry (Figure 2a, b) constructed from *Alnus* and *Salix* shrubs with DRC > D_min_ = 2.5 cm was applied to 1,430 individual tall shrubs measured among 17 circular plots (169 m^2^) in southcentral Alaska. The individual uncertainties were then propagated across the wet field‐mass estimate for each sample‐plot (Figure 2c) by Monte Carlo sampling from lognormal distributions and summing them (Supporting Information). Assuming independence of biomass measures among shrubs in a plot, the plot‐level wet field‐mass for *n* individual shrubs, each with individual prediction error $\varepsilon_{i}$, has uncertainty $U=\sqrt{\sum_{i=1}^{n}\varepsilon_{i}^{2}}$, based on the theorem that the variance of any sum of independent random variables is the sum of the variances (Mendenhall et al., 1981). Thus, applying power function allometry to individual shrubs yields plot‐level uncertainty dominated by the uncertainty of large individuals, as indicated by the very strong, significant correlation between plot‐level uncertainty and count of large shrubs (Figure 2d). A two‐component sampling method reduces the plot‐level uncertainty by reducing the uncertainty of overly influential, large shrubs: those with DRC > *D_max_*. This field protocol minimizes individual shrub uncertainty as measured by the range in the 95% prediction error (95%PI), while increasing the precision and the accuracy of plot‐level biomass estimates. In particular, and by example, we show a substantial increase in precision over the sample plot estimates shown in Figure 2c.

## 2 MATERIALS AND METHODS

Our goal was minimizing uncertainty in biomass estimates given power function allometry. Our protocol considers shrubs as “two‐component” structures (Figure 1): stem internodes of length *L* with *D* > *D_max_* and terminal aerial shoots (tips) with *D_min_* ≤ *D* ≤ *D_max_*, where *D_max_* is a threshold diameter for treating stem internodes as conic frustra and *D_min_* defines the minimum shrub size (e.g., *D_min_* = 2.5 cm). We initially identify *D_max_* subjectively in plots of back‐transformed residuals versus arithmetic values of *D* as the diameter where biomass variability rapidly expands, then adjust *D_max_* if the temporal cost of multiple measurements exceeds the benefit of increased precision. A sketch of how this can be accomplished is presented in the Discussion.

Our allometry example considers only shrub individuals with *DRC* > 2.5 cm = *D_min_* and includes 134 *Alnus* and *Salix* individuals (2.9 ≤ *DRC* ≤ *40*.5 cm; 0.9 ≤ *M* ≤ 374.1 kg) that we destructively sampled in southcentral Alaska to derive biomass‐diameter allometry (Figure 2a, b), shown previously similar for *Alnus* and *Salix* (Lewis‐Clark et al., 2018). Among these shrubs, 29 *Alnus* individuals were dissected and weighed as terminal aerial tips to investigate self‐similarity. We dissected eight further individuals (3.4 ≤ DRC ≤ 36.1 cm; 0.9 ≤ *M* ≤ 374.1 kg) as internodes and terminal aerial tips, each individually weighed and measured.

### 2.1 Field algorithm

Given diameter *D*, single‐component allometric equations describe woody biomass *M* as a power function *M = aD^p^*, with *p* > 2; uncertainty also scales as a power function of *D*, with *p* > 2. The following *two‐component* field‐sampling algorithm reduces uncertainty in estimated biomass of large (*DRC > D_max_*) shrubs, where diameter *D_max_* offers the greatest acceptable uncertainty for *M = aD^p^*.

Step 1: Identify root collar.

Step 2: Record diameter *D_1_* there.

Step 3: If *D_1_* ≤ *D_max_*, stop; aerial tips with *D* ≤ *D_max_* have acceptably low uncertainty. Else if *D_1_* > *D_max_*, identify stem internode above *D_1_* as a conic frustrum. Record its length *L* and end diameters *D_1_* > *D_max_* and *D_2_* (where *D_2_* is measured just below the upper node swelling).

Step 4: Return to Step 2 for stems above the node, treating each stem diameter as *D_1_*.

The individual shrub biomass estimate is the sum of biomass estimates for frustra and aerial tips with associated uncertainties. The uncertainty in each sample‐plot is calculated using Monte Carlo sampling of internodes and tips from lognormal distributions with parameters estimated from log‐log allometry.

*Aerial tip allometry* Inspection of the 134 shrubs indicated that variability in *M* increased substantially at *DRC* > 7.5 cm (Figure 2a), suggesting *D_max_* = 7.5 cm. Using 7.5 cm as the threshold diameter, we established allometric relationships between DRC ≤ *D_max_* and *M* for *n* = 37 individual shrubs; between *D* ≤ *D_max_* and *M* for *n* = 95 terminal aerial tips from large (DRC > *D_max_*); and those two samples taken together (*n* = 132). We also calculated the percent overlap between the aerial tip allometric estimates (including prediction error) and individual shrub allometric estimates. Allometry was established by regressing ln(*M*) on ln(*D*), then exponentiating the 95%PI *upr_i_* and *lwr_i_* bounds of ln(*M_i_*) found with 2.5 ≤ *D_i_* ≤ 7.5 cm at intervals of 0.01 cm to determine uncertainty.

*Stem internode allometry* Stem internodes with *D* > 7.5 cm were modeled as regular conic frustra with diameters *D_1_* and *D_2_* and length *L* (Figure 1b), where frustrum volume *V* = π *L* (*D_1_* ^2^ + *D_1_* *D_2_* + *D_2_* ^2^)/12. We cut, measured, and weighed in the field as wet field‐mass 40 internodes from eight individual alder shrubs of two species (*n* = 20 internodes each from *A. viridus* and *A. incana*). Internodes varied as 2.7 ≤ *D* ≤ 36.1 cm (mean = 9.6 cm), 16.5 ≤ *L* ≤ 224.2 cm, and 0.2 ≤ *M* ≤ 9.1 kg (mean = 8.2 kg). We compared these linear regression wet‐density estimates (i.e., the regression coefficient estimates) to wet‐density directly measured in two field‐cut stem pieces with known weight and estimated volume as measured by submersion in water (*A. incana*: 0.74 kg/L = 3.49 kg/4.73 L and *A. viridis*: 0.83 kg/L = 2.35 kg/2.84 L). Besides regression of untransformed internode variables, we also regressed ln(*M*) on ln(*V*), inspecting both regressions for heteroscedasticity.

### 2.2 Calculating plot‐level uncertainty

The normally distributed error term $\varepsilon_{i}$ (Figure 1) is lognormally distributed and multiplicative when back‐transformed (Kerkhof & Enquist, 2009). Prediction errors (PI) in regression combine the regression error of the estimate with the standard error of the residuals $\sigma_{3}$ and—given an independent variable value—are available for linear models in the statistical package R (vers. 3.6.2; R Core Team 2019) as “*residual.scale*” and “*se.fit*” using the **predict.lm()** function. For *p* > 1, the back‐transformed 95%PI displays positive skew about the expected value *M_i_ = aD_i_^p^* and is bound by

$$lwr_{i}=\exp\left[a‐t_{\left(n‐2,0.975\right)}\sqrt{\text{residual}.{\text{scale}}^{2}+\text{se}.{\text{fit}}^{2}}\right]D_{i}^{b},$$

$$upr_{i}=\exp\left[a+t_{\left(n‐2,0.025\right)}\sqrt{\text{residual}.{\text{scale}}^{2}+\text{se}.{\text{fit}}^{2}}\right]D_{i}^{b}.$$

Given regressions for internodes, aerial tips, and for whole shrubs, we calculated point estimates of wet field‐mass *M* together with upper and lower bounds of 95%PI for every shrub piece and individual measured in the field among 17 sample‐plots (2,019 pieces of 1,430 individual shrubs). Because the sum of lognormal distributions have no closed‐formed distribution (Asmussen & Rojas‐Nandayapa, 2008), we employed the following Monte Carlo algorithm (*n* = 10,000; described in detail in the Supporting Information) to estimate uncertainty for each plot using each sampling method (DRC single‐component and two‐component as tips + internodes):

1. For each shrub or shrub piece in each sample‐plot, estimate the expected_ln(M)_ and se_ln(M)_ using log‐log models fit in R using **lm(),** and *se.fit* = *TRUE* as arguments to the **predict.lm()** function.

2. In the **rlnorm()** function in R, following Bolker (2008) set

$${\text{mean}}_{\text{biomass}}={\;\text{expected}}_{\ln(M)}‐\;\;‐{\text{se}}_{\ln(M)}^{2}/2$$

with

$${\text{sd}}_{\text{biomass}}={\;\text{se}}_{\ln(M)}$$

for all tips and all internodes (and shrubs from one‐component model), then randomly sample from the lognormal distributions *n* times and sum to get *n* Monte Carlo estimates of total sample‐plot biomass.

3. Find the middle 95% quantile of the *n* Monte Carlo samples as a 95%CI of the estimate using the **quantile()** function in R with *p* = *c(0.025,0.975)*.

## 3 RESULTS

### 3.1 Aerial tip allometry

**FIGURE 3:**
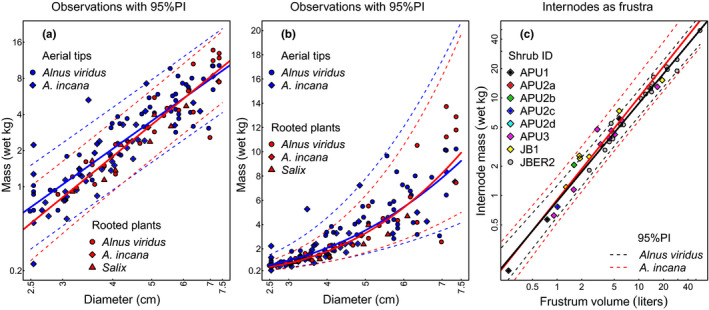
Allometry of two‐component model of shrub biomass. (a) Log of wet field‐mass plotted against diameter for 37 whole shrubs and 95 aerial tips from three taxa (2.5 ≤ *D* ≤ 7.5 cm). Dashed lines indicate 95%PI for aerial tips (blue) and whole plants (red). Solid lines give least squares regression in log‐log space (aerial tips in blue and whole plants in red). (b) Same data and allometry as in (a) but untransformed. (c) Wet field‐mass of alder shrub internodes regressed against internode volume as a frustrum for 40 internodes from eight individual shrubs of two species. The solid lines give best least squares fit for each species (blue: *A. viridus;* red: *A. incana*) with dashed lines the 95%PI

Tall shrubs appear to be self‐similar because the parts and whole share similar allometry (Figure 3). 95%PI for individual alder shrubs with DRC ≤ 7.5 cm nearly overlapped completely (99%) those of aerial tips from alders with DRC > 7.5 cm, suggesting that one regression is sufficient to describe all shrub tips with *D* ≤ 7.5 cm, whether rooted or not (Figure 3ab). While smaller aerial tips tended to be somewhat heavier than rooted plants of the same diameter, the predicted difference for fixed diameter was always less than 0.3 kg across the interval 2.5 ≤ D ≤ 7.5 cm. The ln(*M*) on ln(*D*) regression for combined aerial tips and whole plants with DRC ≤ 10.2 cm was equivalent to *M* = 0.07 *D* ^2.437^ (R^2^ = 0.80, *n* = 133), similar to the whole shrub‐only allometry of *M* = 0.07 *D* ^2.414^ (R^2^ = 0.92, *n* = 134) for individuals with 2.5 ≤ DRC ≤ 40.5 cm (Figure 2b).

### 3.2 Stem internode allometry

Wet field‐mass of alder internodes was well described by internode size as a frustrum volume using simple linear regression (Figure 3c). For *Alnus viridis*, R^2^ = 0.97 (*n* = 20 stems) with a wet‐density estimate of 0.84 ± 0.03 g/cm^3^ (estimate ± se). The directly measured wet‐density (0.83 g/cm^3^ = wet field‐mass/volume) was less than one‐half a standard error of the regression coefficient from the slope estimate. The wet field‐mass of *Alnus incana* was also well fit by frustrum volume (R^2^ = 0.96, *n* = 20 stems); the slope of the regression 0.77 ± 0.04 g/cm^3^ compared favorably to the directly measured wet‐density (0.74 g/cm^3^); the latter was less than one standard error from the point estimate of the slope. Nevertheless, residuals in these regressions visually appeared heteroscedastic in diagnostic plots.

Because log‐transformation of the variables brought about homoscedasticity in residuals, and the difference in wood wet‐density between the two alder species was not significant at the 0.05 level, we regressed ln(*M*) against ln(*V*) ignoring species (Supporting Information). The slope of this log‐log linear regression was essentially equal to one (*p* = 1.0007, se = 0.028), suggesting a linear relationship through the origin. Taking the intercept (*a* = −0.0906, se = 0.057) and exponentiating produced a 95%CI for wet‐density of the stem wood as 0.81 to 1.02 kg/L. We used the log‐log allometric version of internodes in estimating wet field‐mass.

### 3.3 Individual plant‐level uncertainty

Uncertainty—defined as range in 95%PI found with Monte Carlo sampling for individual shrub biomass using single‐component allometry (DRC only) was on average double that using two‐component (tip + internode) allometry when comparing the eight shrubs of known biomass with measured internodes (average calculated exponentiated mean difference of paired log‐uncertainties; *t* = 3.4, *df* = 7, *p* =.006). Individual plant wet field‐mass point estimates from the two models differed by only about 10% on average [geometric mean of ratios = 1.11; two‐tailed paired samples *t*‐test of ln(wet field‐mass) *t* = 1.69, *df* = 7, *p* =.14], as would be expected given that the individual plants were included in constructing both model approaches.

### 3.4 Plot‐level uncertainty

**FIGURE 4:**
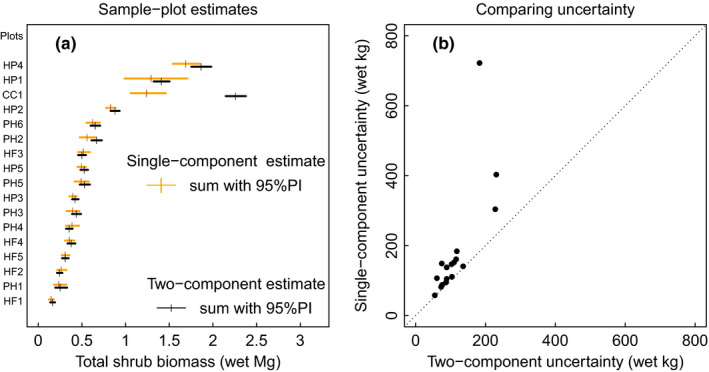
Plot‐level uncertainty using single (DRC only) and two‐component (tip + internode) allometry propagated through shrub individuals to the sample‐plot level. (a) Comparison of the two methods' plot‐level estimates including single‐component estimates shown in Figure 2 (c). Orange vertical ticks give point estimates of summed tall shrub wet field‐mass from single‐component allometry. Vertical black ticks give sum from two‐component allometry (frustra + aerial tips). Horizontal lines give 95%PI. (b) Plot‐level uncertainty in shrub wet field‐mass as difference between upper and lower 95%PI boundaries. Each bullet represents a plot with two wet field‐mass estimates: single‐ and two‐component. Single‐component uncertainties are generally greater than or equal to two‐component allometry uncertainties. Dotted line has slope of one and passes through origin

At the plot‐level, the single‐component uncertainties (Figure 4b) were on average 39% more than two‐component uncertainties and significantly so (one‐tailed paired samples *t*‐test of ln(uncertainty): *t* = 4.07, *p* =.0004, *df* = 16). Point estimates of total shrub wet field‐mass in sample plots (Figure 4a) were generally smaller in single‐component than in two‐component models (paired samples *t*‐test of logged point estimates *t* = −2.5, *p* =.023, *df* = 16; Figure 4a).

Overall, for individual shrubs with DRC > *D_max_* the single‐component uncertainty was halved by the two‐component method and accuracy increased. At the plot‐level, we found that among 1,430 individual *Salix* and *Alnus* shrubs (2.5 ≤ DRC ≤ 30.4 cm) measured in 17 plots (169m^2^), the uncertainty in total sample‐plot biomass estimation using the two‐component method was 40% less than the single‐component method; this difference depended on the count of shrubs with DRC > *D_max_*.

## 4 DISCUSSION

Increases in shrubs in arctic and dryland ecosystems are well‐documented but poorly quantified as measured change in biomass. Part of the reason is a lack of allometry. Because these landscapes of increasing tall shrubs are often vast and remote, quantifying abundance there relies on remote sensing informed by fieldwork at the plot‐level (Greaves et al. 2016, Berner et al., 2018; Alonzo et al., 2020) that applies allometry. Scaling from the individual to the landscape and beyond requires plant‐level measures that *precisely* estimate plot‐level biomass to inform landscape‐level models with error propagation. The propagation of error in remote‐sensing informed biomass estimates is an increasingly important consideration. That importance motivates this study.

We found three results pertinent to the problem of reducing uncertainty in estimates of tall shrub biomass based on allometric power functions of stem diameter *D*. First, uncertainty of allometry for aerial stem tips (see Figure 1) overlapped the allometry for rooted shrubs. This result of self‐similarity—that one allometric equation is sufficient (Figure 2a, b) for a given taxon's parts and whole—is a remarkable and useful application of fractal geometry, because it means that parts of shrubs can be measured for biomass using whole shrub allometry. One reason that field practitioners should take note of this result is that large shrubs have the greatest uncertainty both for statistical and for life‐history reasons: older shrubs accumulate damage as well as opportunistic growth. These large shrubs contribute to the greatest uncertainty in plot‐level biomass estimates due to the nonlinear nature of allometry. By applying allometry to the components of large shrubs, particularly their internode stems which show far less uncertainty than tips, whole‐shrub uncertainty is substantially reduced.

A second valuable result for those of us interested in estimating tall shrub biomass is that stem internode biomass fits well a simple conic frustral volume using log‐log linear regression with a regresion slope of one. This result has the benefit of passing through the origin as a linear function with log‐normally distributed residuals. Supporting the use of frustra as geometric models of stem internodes (Figure 2c), independently measured field‐wet wood density was statistically indistinguishable from the regression coefficient of field‐mass on frustrum volume. The calculation of a frustum volume is simple and requires only three measurements. We found that measuring a frustrum's three dimensions took roughly four times as long as measuring a single diameter; however, it can greatly reduce the uncertainty in shrub biomass estimates, particularly when taper is rapid.

Finally, we note that the application of the two‐component method of measuring shrubs as internodes plus terminal aerial tips can reduce whole‐shrub uncertainty by more than 50% over single‐component estimates and reduce plot uncertainty by 40% (Figure 3). The reduction increases with abundance of large diameter shrubs in field plots. Of course, this reduction can be an even greater reduction by choosing *D_max_* near the minimum size of shrub stems; however, the smaller the cutoff, the more stems per shrub must be measured, increasing sampling time.

### 4.1 Finding D_max_ and the cost to apply it

The cutoff diameter measure *D_max_* is a critical value best found from (1) project‐specific allometry (allometry is notoriously regional; Chojnacky & Milton, 2008); (2) an estimate of the distribution of stem diameters; and (3) empirical knowledge of sampling times. The value of *D_max_* may also be obvious from a visual plot of the absolute value of residuals (i.e., variability = |residuals|) versus DRC as a diameter near where variability in biomass increases dramatically. It is convenient to choose an integer value to increase speed of measurement in the field (e.g., here *D_max_* = 7.5 cm = 3 inches). To justify taking more measurements, the increase in precision of allometric prediction must outweigh the cost of increased measurement time.

Given the allometry shown in Figure 2b, the prediction error (defined as the difference between upper prediction and lower prediction values) scales approximately as *D* ^2.4^ [as found by regressing log(prediction error) on log(diameter)], showing clearly that as *D_max_* approaches zero, so does the uncertainty in biomass estimate. A more rigorous approach than visual inspection of the raw data, is to choose an initial value for *D_max_* from the untransformed allometry and apply it in the field in pilot sampling, so as to estimate the time required to make single component measurements versus two‐component ones. The ratio of time taken for two component measures to single component measures can then be compared to the ratio of expected single‐component prediction error to two‐component prediction errors using *D_max_*.

To calculate the expected prediction error (E[*PE*]) across all diameters requires both allometry of prediction error (e.g., here *PE* = *k D* ^2.4^, where *k* = exp(−2.02) is the scaling factor) and a probability density function of stem diameters, pdf(*D*), such that the expected prediction error for a single‐component method of a single randomly sampled shrub from pdf(D) is

(1) $$E\left[PE_{1}\right]=\int_{\mathrm{smallestdiameter}}^{\mathrm{largestdiameter}}\text{pdf}\left(x\right)kx^{2.4}dx$$

where the integral is taken over all diameters possibly measured. For the two‐component method (ignoring the frustum error which is much smaller than the tip error), the expected prediction error of a single shrub measurement is

(2) $$E\left[PE_{2}\right]=\int_{\mathrm{smallestdiameter}}^{\mathrm{Dmax}}\text{pdf}\left(x\right)kx^{2.4}dx.$$

If the ratio of expected prediction error for a single‐component to prediction error for two‐component (*i.e*., $E\left[PE_{1}\right]/E\left[PE_{2}\right]$) is greater than or equal to the ratio of expected time for two‐component measure to time for single‐component measure (*i.e*., ($E\left[t_{2}\right]+t_{1})/t_{1}$), then applying a two‐component strategy with *D_max_* improves precision by a factor more than or equal to its cost in time: $E[PE_{1}]/E[PE_{2}]\geq (E[t_{2}]+t_{1})/t_{1}.$

### 4.2 Worked example

We provide an example of calculating times per measurement from actual field data with *D_max_* = 10 cm for alders among *n* = 14 field plots (7.25 m radius) sampled during one day on Alaska's Kenai Peninsula. Using measurements of 1,286 tips and 87 frustra over a total of 14.7 person‐hours spent in within field plots, we applied a multiple linear regression as *total time per plot* = *t_0_* + *t_1_* * (*count of single‐component measures per plot*) + *t_2_* * (*count of two‐component measure per plot*) + ε(0,σ), finding *R^2^* = 0.96, ε ~ *N*(0,543), and *t_1_* = 26 s as *mean time of single component measure* and *t_2_* = 111 s as *mean time of two‐component measure*. To calculate the expected time given the stem diameter distribution for the two‐component measure, E[*t*_2_], requires knowing the proportion of stems with diameters >*D_max_* =10 cm (call this proportion *P_D>Dma_*_x_) to calculate E[*t*_2_] = *t*_2_ *P_D>Dma_*_x_ to which the single‐component value *t_1_* must also be added, because given a stem it is first measured to determine if it must be sampled as a frustrum. Hence the addition of *t_1_* to *t*_2_ weighted by the probability that its diameter is greater than *D_max_*.

To approximate the integrals given by Equations (1) and (2), we constructed a kernel density of the distribution of stem diameters from one day of sampling (n = 1,286 stems) using function **density()** with its defaults in base R; the output of **density()** includes the random variable as *x* (here diameter) and the density value as *y*. We approximated the pdf in Equation (1) using the default *n = *512 diameters for *x* (range: 0.85–18.5 cm) estimated by **density(),** calculated the prediction error at each *x_i_* using the allometry $PE_{1}=\exp\left(‐2.02\right)D^{2.41}$, then multiplied each prediction error $PE_{1}$ by the density estimate *y_i_* at each *x_i_*, summed and multiplied by Δ*x* = (18.5–0.85)/512 to arrive at

$$E\left[PE_{1}\right]=\sum_{i=1}^{512}y_{i}\exp\left(‐2.02\right)x_{i}^{2.41}x=14.6\text{kg}.$$

expected error per measurement. Using *D_max_* = 10 cm = *x_266_* and approximating Equation (2) as

$$E\left[PE_{2}\right]=\sum_{i=1}^{266}y_{i}\exp\left(‐2.02\right)x_{i}^{2.41}x=10.4\text{kg}.$$

Their ratio is $E\left[PE_{1}\right]/E\left[PE_{2}\right]=1.4$.

Weighting the time of sampling stems requiring the two‐component method (111 s) by the proportion of stems with *D* ≥ *D_max_* = 10 cm (here, *P_D>Dma_*_x_ = 0.09) gives E[*t*_2_] = (111 s) × (0.09) = 10.0 s and adding *t_1_* = 26 s gives 36 s. The ratio of expected time for a two‐component measure to a single‐component measure is 36/26 = 1.38 ≤ 1.4 = $E\left[PE_{1}\right]/E\left[PE_{2}\right]$, suggesting that *D_max_* is well‐chosen, because the cost in time of a two‐component measure is less than or equal to its benefit of reduction in uncertainty.

In summary, to rigorously calculate an optimal *D_max_* requires allometry with known prediction errors, some prior knowledge of the distribution of stem diameters, and an estimate of the time required for both single‐component and two‐component measurements.

## CONFLICT OF INTEREST

As authors, we declare no conflict of interest in the publication of this study.

## AUTHOR CONTRIBUTIONS

**Roman Dial:** Conceptualization (equal); Data curation (lead); Formal analysis (lead); Writing‐original draft (lead). **Bethany Schulz: Conceptualization (equal);** Investigation (equal); Methodology (supporting); Supervision (lead); Writing‐review & editing (equal). **Eric Lewis‐Clark:** Data curation (equal); Formal analysis (equal); Investigation (equal); Methodology (equal); Writing‐review & editing (equal). **Kaili Martin:** Investigation (equal); Methodology (equal); Writing‐review & editing (equal). **Eric Anderson:** Funding acquisition (equal); Investigation (equal); Project administration (equal); Resources (equal); Supervision (equal); Writing‐review & editing (equal).

## Supporting information

Supplementary Material

## Data Availability

Our datasets reside in archive at Dryad as: Dial, Roman; Anderson, Hans‐Eric; Martin, Kaili; Schulz, Bethany (2020), Tall Shrub Biomass Estimates, *Dryad*, Dataset, doi.org/10.5061/dryad.6hdr7sqzn.

## References

[ece37393-bib-0001] Alonzo, M. , Dial, R. J. , Schulz, B. K. , Andersen, H. E. , Lewis‐Clark, E. , Cook, B. D. , & Morton, D. C. (2020). Mapping tall shrub biomass in Alaska at landscape scale using structure‐from‐motion photogrammetry and lidar. Remote Sensing of Environment, 245. 111841.– 10.1016/j.rse.2020.111841

[ece37393-bib-0002] Asmussen, S. , & Rojas‐Nandayapa, L. (2008). Asymptotics of sums of lognormal random variables with Gaussian copula. Statistics & Probability Letters, 78, 2709–2714. 10.1016/j.spl.2008.03.035

[ece37393-bib-0003] Berner, L. T. , Alexander, H. D. , Loranty, M. M. , Ganzlin, P. , Mack, M. C. , Davydov, S. P. , & Goetz, S. J. (2015). Biomass allometry for alder, dwarf birch, and willow in boreal forest and tundra ecosystems of far northeastern Siberia and north‐central Alaska. Forest Ecology and Management, 337, 110–118. 10.1016/j.foreco.2014.10.027

[ece37393-bib-0004] Berner, L. T. , Jantz, P. , Tape, K. D. , & Goetz, S. J. (2018). Tundra plant above‐ground biomass and shrub dominance mapped across the North Slope of Alaska. Environmental Research Letters, 13. 035002.– 10.1088/1748-9326/aaaa9a

[ece37393-bib-0005] Bolker, B. M. (2008). Ecological models and data in R. Princeton. Princeton University Press.

[ece37393-bib-0006] Chojnacky, D. C. , & Milton, M. (2008). Measuring carbon in shrubs. In C. M. Hoover (Ed.), Field measurements for forest carbon monitoring (pp. 45–72). Springer Science & Business Media.

[ece37393-bib-0007] Conti, G. , Gorné, L. D. , Zeballos, S. R. , Lipoma, M. L. , Gatica, G. , Kowaljow, E. , Whitworth‐Hulse, J. I. , Cuchietti, A. , Poca, M. , Pestoni, S. , & Fernandes, P. M. (2019). Developing allometric models to predict the individual aboveground biomass of shrubs worldwide. Global Ecology and Biogeography, 28, 961–975. 10.1111/geb.12907

[ece37393-bib-0502] Greaves, H. E. , Vierling, L. A. , Eitel, J. U. , Boelman, N. T. , Magney, T. S. , Prager, C. M. , & Griffin, K. L. (2016). High‐resolution mapping of aboveground shrub biomass in Arctic tundra using airborne lidar and imagery. Remote Sensing of Environment, 184, 361–373.

[ece37393-bib-0008] Kerkhoff, A. J. , & Enquist, B. (2009). Multiplicative by nature: Why logarithmic transformation is necessary in allometry. Journal of Theoretical Biology, 257, 519–521. 10.1016/j.jtbi.2008.12.026

[ece37393-bib-0009] Lewis‐Clark, E. , Dial, R. , & Schulz, B. (2018). Developing remotely sensed methods for estimating tall shrub biomass in forest and subalpine communities: Linking plot‐level measures to LiDAR. In S. P. Healey & V. M. Berrett (Eds.) Doing more with the core: Proceedings of the 2017 forest inventory and analysis (FIA) science stakeholder meeting; 2017 October 24‐26; Park City, Utah (pp. 33‐37). USDA Forest Service.

[ece37393-bib-0010] Mendenhall, W. , Scheaffer, R. L. , & Wackerly, D. D. (1981). Mathematical statistics with applications, (2nd ed.). Duxbury Press.

[ece37393-bib-0011] Paul, K. , Roxburgh, S. , Chave, J. , England, J. , Zerihun, A. , Specht, A. , Lewis, T. , Bennett, L. T. , Baker, T. G. , Adams, M. A. , Huxtable, D. , Montagu, K. D. , Falster, D. S. , Feller, M. , Sochacki, S. , Ritson, P. , Bastin, G. , Bartle, J. , Wildy, D. , … Sinclair, J. (2015). Testing the generality of above‐ground biomass allometry across plant functional types at the continent scale. Global Change Biology, 22, 2106–2124. 10.1111/gcb.13201 26683241

[ece37393-bib-0501] R Core Team (2019). R: A language and environment for statistical computing. Vienna, Austria: R Foundation for Statistical Computing. https://www.R-project.org/

[ece37393-bib-0012] Woodall, C. W. , Heath, L. S. , Domke, G. M. , & Nichols, M. C. (2011). Methods and equations for estimating aboveground volume, biomass, and carbon for trees in the US forest inventory, 2010. General Technical Report NRS‐88. USDA Forest Service.

[ece37393-bib-0013] Xiao, X. , White, E. P. , Hooten, M. B. , & Durham, S. L. (2011). On the use of log‐transformation vs. nonlinear regression for analyzing biological power laws. Ecology, 92, 1887–1894. 10.1890/11-0538.1 22073779

