# Supplementary material for: Using fractal self‐similarity to increase precision of shrub biomass estimates
Source: Ecol Evol. 2021 Mar 18;11(9):4866–73. doi: 10.1002/ece3.7393 (PMC8093737; doi:10.1002/ece3.7393)
Supplement: Supplementary file 1 — Supplementary Material [file ECE3-11-4866-s001.pdf]

Supplementary Information: Propagating sums of lognormal errors generated from power-law allometry

Dial, RD, Martin, K, Schulz, B, Lewis-Clark, E, and Andersen H

September 2, 2020

We have developed a field protocol for collecting shrub biomass where shrubs are seen as self-similar in their aerial branching architecture. The idea is two-part. First, the same allometric relationship between biomass of a stem and stem diameter holds for "small" shrubs and aerial parts of "large" shrubs, where "small" shrubs have a basal diameter (diameter at root collar, DRC) less than some defined threshold (D_{max}) and "large" have $DRC \geq D_{max}$. The second part of the protocol assumes that shrub internodes that have diameters $D \geq D_{max}$ are well described as conic frustra.

These notes and code address the question, "How should the uncertainty from the individual pieces of shrubs be propagated into the *sum of all pieces* in a field plot?" The question is answered in the context of a similar treatment using a more standard, single-component approach where DRC alone is used to estimate shrub biomass.

Biomass Allometry

Here are the models we use for estimating shrub field-mass (wet.kg) using stem diameters and internode lengths for tall shrubs (*Alnus viridis* and *A. incana*: Betulaceae and *Salix spp.*: Salicaceae) in southcentral Alaska.

Single-component Shrub Biomass Allometry

This model uses the DRC alone to estimate biomass:

```
suppressMessages(suppressWarnings(library(tidyverse)))
load("mod.RData")
summary(mod)
```

```
##
## Call:
## lm(formula = log(wet.mass.kg) ~ log(diam.cm), data = indiv)
##
## Residuals:
##      Min       1Q   Median       3Q      Max
## -1.32936 -0.28683  0.04998  0.30580  0.77436
##
## Coefficients:
##              Estimate Std. Error t value Pr(>|t|)
## (Intercept)  -2.70769    0.14700  -18.42   <2e-16 ***
## log(diam.cm)  2.41350    0.06219   38.81   <2e-16 ***
## ---
## Signif. codes:  0 '***' 0.001 '**' 0.01 '*' 0.05 '.' 0.1 ' ' 1
##
```

```
## Residual standard error: 0.4276 on 132 degrees of freedom
## Multiple R-squared:  0.9194, Adjusted R-squared:  0.9188
## F-statistic:  1506 on 1 and 132 DF,  p-value: < 2.2e-16
```

The log-log linear regression, with an $R^2 = 0.92$, above is equivalent to the power function

$$M = e^{-2.70769}\ \text{DRC}^{2.4135} = 0.07\ \text{DRC}^{2.414}.$$

While not ideal, we'll use a subset of the data used to construct the model above to calculate RMSE and 95%PI of wet-mass. Later, we'll do the same for the two-component model and compare them for accuracy and precision, recognizing that these are data from the training dataset.

```
dat8 <- read.csv("../Data/AldersDissected.cm.csv")
str(dat8)
```

```
## 'data.frame':    95 obs. of  5 variables:
##  $ plant.id   : Factor w/ 8 levels "APU1","APU2a",..: 1 1 1 1 1 1 1 1 2 3 ...
##  $ wet.mass.kg: num  6.2 0.6 0.2 0.7 0.5 1.9 2.1 1.5 1.2 2.1 ...
##  $ diam.cm    : num  9.7 3.9 3.3 2.7 3.1 3.9 4.3 4 3.4 5.3 ...
##  $ diam2.cm   : num  5.6 2.7 3.1 0 0 0 0 0 0 4.5 ...
##  $ length.cm  : num  123.2 88.3 31.8 0 0 ...
```

Here it is aggregated into individual shrubs:

```
wet.mass8.kg <- aggregate(wet.mass.kg~plant.id,FUN=sum,data=dat8)
wet.mass8.kg$diam.cm <- aggregate(diam.cm~plant.id,FUN=max,data=dat8)$diam.cm
wet.mass8.kg$pieces.count <- aggregate(wet.mass.kg~plant.id,FUN=length,data=dat8)$wet.mass.kg
wet.mass8.kg <- wet.mass8.kg [order(wet.mass8.kg$wet.mass.kg),]
rownames(wet.mass8.kg) <- 1:8
wet.mass8.kg
```

```
##   plant.id wet.mass.kg diam.cm pieces.count
## 1    APU2a         1.2     3.4            1
## 2    APU2d         1.4     4.0            1
## 3    APU2c         3.1     4.7            3
## 4    APU2b         3.7     5.3            2
## 5     APU1        13.7     9.7            8
## 6      JB1        42.5    12.2           13
## 7     APU3        67.2    15.8           20
## 8    JBER2       382.2    36.1           47
```

Using the **predict.lm()** function with *se.fit=TRUE* to get wet-mass estimates from these eight alders using DRC;

```
drc.alders.pred.log.kg <- predict(mod,newdata=wet.mass8.kg,interval="prediction") %>%
  data.frame()

drc.alders.pred.log.kg$plant.id <- wet.mass8.kg$plant.id
drc.alders.pred.log.kg$mass.kg <- wet.mass8.kg$wet.mass.kg
drc.alders.pred.log.kg$drc.est.kg <- round(exp(drc.alders.pred.log.kg$fit),1)
drc.alders.pred.log.kg$drc.err.kg <-
  round(exp(drc.alders.pred.log.kg$fit)-wet.mass8.kg$wet.mass.kg,1)
drc.alders.pred.log.kg$drc.lwr.kg <- round(exp(drc.alders.pred.log.kg$lwr),1)
drc.alders.pred.log.kg$drc.upr.kg <- round(exp(drc.alders.pred.log.kg$upr),1)
drc.alders.pred.log.kg$drc.unc.kg <-
  round(exp(drc.alders.pred.log.kg$upr)-exp(drc.alders.pred.log.kg$lwr),1)
```

```r
(drc.alders.pred.log.kg <- drc.alders.pred.log.kg[,c("plant.id","mass.kg",
                                                   "drc.est.kg","drc.err.kg","drc.lwr.kg",
                                                   "drc.upr.kg","drc.unc.kg")])
```

```
##   plant.id mass.kg drc.est.kg drc.err.kg drc.lwr.kg drc.upr.kg drc.unc.kg
## 1    APU2a     1.2        1.3        0.1        0.5        3.0        2.5
## 2    APU2d     1.4        1.9        0.5        0.8        4.5        3.7
## 3    APU2c     3.1        2.8       -0.3        1.2        6.6        5.4
## 4    APU2b     3.7        3.7        0.0        1.6        8.8        7.2
## 5     APU1    13.7       16.1        2.4        6.9       37.5       30.7
## 6      JB1    42.5       27.9      -14.6       11.9       65.3       53.4
## 7     APU3    67.2       52.1      -15.1       22.3      122.1       99.8
## 8    JBER2   382.2      382.9        0.7      161.4      908.4      747.0
```

Two-component Shrub Biomass Allometry

This is the new, two component approach with one allometric equation for aerial tips based on diameter and a second for internodes based on their volume as approximated by a conic frustrum.

Internode allometry If each internode is considered as a conic frustrum with volume calculated using the length of the internode (L) and its two end diameters (D_i,) then volume is

$$V = \pi L[D_1^2 + D_2^2 + (D_1 D_2)]/12.$$

Previous analysis shows that field mass can be regressed on volume in a linear fashion with a slope very close to the measured density of species-specific alder wood; however, the residuals around the best-fit line are unequal across field-masses. The log-log regression is homoscedastic in residuals and so is preferred. The log-log regression also has the nice feature that its regression coefficient, i.e., the slope p equivalent to the power on volume, is essentially one (see below), indicating that it is a linear relationship between an internode's mass and volume as frustrum; the intercept of the log-log regression a as a power of e is the wood's wet density: density $= e^a$.

```r
load("frustrum.mod.RData")
summary(frustrum.mod)
```

```
##
## Call:
## lm(formula = log(wet.kg) ~ log(volume.liter), data = dat.cylinder)
##
## Residuals:
##      Min       1Q   Median       3Q      Max
## -0.34861 -0.14606 -0.02703  0.09667  0.51722
##
## Coefficients:
##                   Estimate Std. Error t value Pr(>|t|)
## (Intercept)       -0.09060    0.05730  -1.581    0.122
## log(volume.liter)  1.00071    0.02822  35.459   <2e-16 ***
## ---
## Signif. codes:  0 '***' 0.001 '**' 0.01 '*' 0.05 '.' 0.1 ' ' 1
##
## Residual standard error: 0.2042 on 38 degrees of freedom
## Multiple R-squared:  0.9707, Adjusted R-squared:  0.9699
## F-statistic:  1257 on 1 and 38 DF,  p-value: < 2.2e-16
```

So the log-log regression of field-mass ($frust$) on V is essentially

$$frust = e^a V^p$$

where $e^a = e^{-0.09060} = 0.91$ kg L^{-1} is an estimate of the density of the wet alder wood and $p = 1.00071 \approx 1$, so that

$$frust = e^{-0.09060} V^{1.0071} \approx 0.91V.$$

This linear allometric relation has the nice feature that it passes naturally through the origin and offers a high $R^2 = 0.97$ without forcing through the origin. The residuals of the log-log regression are normally distributed and so lognormal on the arithmetic scale.

Aerial tip allometry For the aerial branching tips, where $D_{max} = 10.2$ cm $= 4$ inches we use:

```
load("all.under7.5.RData")
summary(all.under7.5)
```

```
##
## Call:
## lm(formula = log(wet.kg) ~ log(diam.cm), data = under.7.5cm)
##
## Residuals:
##      Min       1Q   Median       3Q      Max
## -1.10044 -0.22336 -0.02619  0.24087  1.30159
##
## Coefficients:
##              Estimate Std. Error t value Pr(>|t|)
## (Intercept)   -2.6504     0.1597  -16.60   <2e-16 ***
## log(diam.cm)   2.4149     0.1057   22.84   <2e-16 ***
## ---
## Signif. codes:  0 '***' 0.001 '**' 0.01 '*' 0.05 '.' 0.1 ' ' 1
##
## Residual standard error: 0.3784 on 130 degrees of freedom
## Multiple R-squared:  0.8005, Adjusted R-squared:  0.799
## F-statistic: 521.7 on 1 and 130 DF,  p-value: < 2.2e-16
```

The log-log regression is mathematically equivalent to the power function,

$$tip = e^a D^p = e^{-2.6504} D^{2.4149} \approx 0.07\ D^{2.415}$$

where tip is field-mass, D diameter, with $a = -2.6504$ and $p = 2.4149$ the intercept and regression slope, the latter an allometric scaling constant within 0.8 standard error units of 2.5, a multiple of 0.25 considered a universal scaling multiple (West 2017). Again, the residuals of the log-log regression are normally distributed and so lognormal on the arithmetic scale.

Lognormal Distributions and Statistics of Sums

Ben Bolker's *Ecological Models and Data in R* (page 137) and Wikipedia's Lognormal entry show that if Y is a random variable distibuted lognormally, LN, as $Y \sim LN(\mu, \sigma)$ where $\mu = E[\ln Y]$ is the mean of the logarithm of Y, and $\sigma = \sqrt{E[\ln Y^2]}$ is the standard deviation of the logarithm of Y, then

$$var(y) = e^{2\mu+\sigma^2}(e^{\sigma^2} - 1)$$

and

$$mean(y) = \hat{y} = e^{\mu + (\sigma^2)/2} = e^{\mu}\ e^{\sigma^2/2}$$

or

$$\ln(\hat{y}) = \mu + \sigma^2/2$$

rearranged as

$$\mu = \ln(\hat{y}) - \frac{\sigma^2}{2}.$$

So in terms of a regression involving lognormal residuals, the expected value of field-mass is $E[Y] = \hat{y}$ with $\ln(E[Y]) = \ln(\hat{y})$ meaning the distribution of y in R can be represented as the predicted fit value minus one half the standard error of the residuals.

We know from basic statistical theory (Mendenhall, Sheaffer, and Wackerly 1981) that given any sum of random variables $W = \sum_i^m X_i$, the mean of the sum is the sum of the means,

$$\bar{W} = E[W] = \sum_i^m \bar{X}_i = \sum_i^m E[X_i]$$

and the variance of the sum is the sum of the variances

$$var(W) = \sum_i^m var(X_i).$$

Thus, given $s = T + F$ pieces of shrubs with T tips and F internodes in a plot, where each tip has expected mass given by the tip allometry (mass $= \hat{tip}$) and internodes have mass as density times the frustral volume (mass $= \hat{frust}$), then we sum the field-masses to get the total mass in a plot and sum the variances to get the variance. Apparently, the sum of lognormal distributions have no closed-formed distibution (Asmussen and Rojas-Nandayapa, 2009), so we simulate the distribution with Monte Carlo sampling.

The mean is straightforward;

$$\bar{W} = \bar{W}_{tips} + \bar{W}_{frust} = \sum_{i=1}^T \hat{tip}_i + \sum_{i=1}^F \hat{frust}_i = \sum_{i=1}^T e^a D_i^p + \sum_{i=1}^F e^b D_i^c$$

The variance is messier than the mean, but conceptually simple. Given one field measurement used to predict field-mass, the uncertainty in that predicted mass arises from both regression uncertainty in the parameters and from the uncertainty around the regression model (residuals). That is, the variance of sum of the masses depends on each model prediction, that in turn equals the sum of the regression variance (se^2_{reg}, dependent on the tip diameter or internode volume distance from their mean value used to construct the regression) and the residual variance (σ^2).

For an individual internode, the prediction variance depends on the volume V and the regression statistics (residuals variance $= \sigma^2$, sample size $= n$, mean volume $= \bar{V}$, variance of volumes $= s_V^2$) with

$$var(w_{pred}) = \hat{\sigma}^2 + se^2_{reg}$$

and

$$se^2_{reg} = \frac{\hat{\sigma^2}}{n} + \frac{\hat{\sigma}^2(V - \bar{V})}{s_V^2(n-1)}$$

Approach using R

To combine the two terms in the variance estimate for any given sampling situation, the approach here uses the base R function **predict.lm()** with argument **se=TRUE.** This provides both σ as **residual.scale** and se_{reg} as **se.fit** in the output of the predict function. Thus the variance in the prediction estimate for any single predicted field-mass is the sum of the squared value of **residual.scale** and the squared value of **se.fit.** That is,

$$var(w_{pred}) = \hat{\sigma}^2 + se_{reg}^2 = \texttt{residual.scale}^2 + \texttt{se.fit}^2$$

A 95% confidence interval for log-log regresions such as for tips would be:

$$95\% \log(\text{CI}) = a + p\log(D) \pm t_{n-2}(0.975)\sqrt{\texttt{residual.scale}^2 + \texttt{se.fit}^2}$$

but in arithmetic space the lower limit is

$$95\%(\text{CI})_{lwr} = e^{a - t_{n-2}(0.975)\sqrt{\texttt{residual.scale}^2 + \texttt{se.fit}^2}} D^p$$

with the upper limit as

$$95\%(\text{CI})_{lwr} = e^{a + t_{n-2}(0.975)\sqrt{\texttt{residual.scale}^2 + \texttt{se.fit}^2}} D^p,$$

from which it's clear how error increases as a power function of diameter D with $p \geq 1$. However, rather than use these formulas, which work well for single-component allometry and are conveniently calculated by **predict.lm()**, we'll use a Monte Carlo approach because we need to sum lognormally distributed random variables, and then find the distributions of these sums (which have no closed-form solution). We need this both for individual shrubs and sample-plots.

The recipes above give individual shrub uncertainities and their sums as plot-level uncertainties. But these first moments still beg the question of what distribution is the sum of all these lognormal distributions as the total weight W? Even with the mean and the variance in hand, we still are not clear what distribution to sample from when we pass on the plot-level uncertainty to the lidar-assisted estimate of, say, landscape estimates of shrub biomass.

Approximating Individual Shrub Biomass Distributions

Again we'll use the eight alder dataset, but this time get each piece's estimate as expected log(we.mass), sd(residuals), and se(fit), then use a Monte Carlo method to estimate the distribution of each shrub as sum of lognormal distributions of tip and internode pieces.

1. Find the expected mean and se of each shrub piece's biomass recognizing that the field-mass of tip and internode estimates are lognormally distributed.
2. For tips and internodes, sample from lognormal distributions 10,000 times with mean given by the expected log of field-mass and prediction uncertainty given by $\texttt{residual.scale}^2 + \texttt{se.fit}^2$.
3. For each of the 10,000 simulations, sum each individual shrub's pieces, find the mean, and the 0.025 and 0.975 quantile to give the point estimate and an estimate of propagated error.

One-component allometry estimates for whole shrubs using Monte Carlo simulation

For fair comparison we'll apply the Monte Carlo method to the single-compoinent allometry as well. First apply the single-component allometry.

```
drc.8.kg.pred <- predict(mod,
                         newdata=wet.mass8.kg,
                          se.fit=T) %>% data.frame()
```

```
drc.8.kg.pred$var.kg <- drc.8.kg.pred$se.fit^2 + drc.8.kg.pred$residual.scale^2
drc.8.kg.pred$plant.id <- wet.mass8.kg$plant.id
```

Next simulate sampling with a Monte Carlo Approach (I know this could be done much simpler using vetorization, but just wantto check that same method applied for each modeling approach to avoid artifacts of methods.)

```
# n random simulations
n <- 10000
rando <- 1:n ## one shrub simulation

## shrub individuals
shrubs.id <- levels(drc.8.kg.pred$plant.id)
shrub.count <- length(shrubs.id) ## how many shrubs

## vectors to hold results
lwr.shrub <- 1:shrub.count
upr.shrub <- 1:shrub.count
expcted.shrub <- 1:shrub.count

## loop over shrubs
for(i in 1:shrub.count){
    whole.shrub <- subset(drc.8.kg.pred,
                        plant.id==shrubs.id[i])
## loop over simulations as one for every shrub
## but do so n times, saving the total mass in kg
  for(j in 1:n){ ## sample each shrub n times
      mean.log.kg <- whole.shrub$fit ## the expected value shrub biomass
      se.log.kg <- sqrt(whole.shrub$var.kg) ## the standard error of tips
    ## sample each tip randomly from "lognormal" being sure to adjust lognormal mean
    shrub.samples <- rlnorm(1,
                    mean.log.kg-(se.log.kg^2)/2, ## important!
                    se.log.kg)
      rando[j] <- shrub.samples
  }
    ## Find the 95% quantile endpoints and expectation for each shrub
 lwr.shrub[i] <- quantile(rando,p=0.025,na.rm=T)
 upr.shrub[i] <- quantile(rando,p=0.975,na.rm=T)
 expcted.shrub[i] <- mean(rando,na.rm=T)
}

## save summaries as dataframe
drc.dat8.est <- data.frame(plant.id=shrubs.id,
                 drc.est.kg=round(expcted.shrub,1),
                 drc.lwr.kg=round(lwr.shrub,1),
                 drc.upr.kg=round(upr.shrub,1),
                 drc.unc.kg=round(upr.shrub-lwr.shrub,1))
## display
drc.dat8.est <- drc.dat8.est[order(drc.dat8.est$drc.est.kg),]
rownames(drc.dat8.est) <- 1:dim(drc.dat8.est)[1]
drc.dat8.est
```

```
##   plant.id drc.est.kg drc.lwr.kg drc.upr.kg drc.unc.kg
## 1    APU2a        1.3        0.5        2.7        2.2
## 2    APU2d        1.9        0.7        3.9        3.2
```

```
## 3    APU2c        2.8         1.1         6.0         4.9
## 4    APU2b        3.7         1.5         7.8         6.4
## 5     APU1       16.0         6.3        34.2        27.9
## 6      JB1       27.8        11.0        59.0        48.0
## 7     APU3       52.3        20.7       108.8        88.1
## 8    JBER2      381.8       149.0       797.6       648.6
```

Two-component allometry for whole shrubs with propagation of uncertainty in tips and internodes

First we prepare the **dat8** file for tips and node allometry.

```
dat8$volume.liter <- with(dat8,
   ((pi/3)*length.cm*((diam.cm/2)^2 + (diam2.cm/2)^2 + (diam2.cm/2)*(diam.cm/2))))/1000

dat8 <- dat8[,c("plant.id","volume.liter","diam.cm")]
```

First apply the two component allometry, for tips:

```
dat8.tips <- subset(dat8,volume.liter == 0)
pred.log.tips8.kg <- predict(all.under7.5,
  newdata=dat8.tips,
  se=T) %>% data.frame()

pred.log.tips8.kg$var.log <- pred.log.tips8.kg$se.fit^2 + pred.log.tips8.kg$residual.scale^2

pred.log.tips8.kg$plant.id <- dat8.tips$plant.id
```

And for internodes (also logged but not well named variables):

```
dat8.frust <- subset(dat8,volume.liter != 0)

pred.frust8.kg <- predict(frustrum.mod,
  newdata=dat8.frust,
  se=T) %>% data.frame()

pred.frust8.kg$var.kg <- pred.frust8.kg$se.fit^2 + pred.frust8.kg$residual.scale^2

pred.frust8.kg$plant.id <- dat8.frust$plant.id
```

Shrub estimates:

```
# n random simulations
n <- 10000
rando <- 1:n ## one shrub simulation

## shrub individuals
shrubs.id <- levels(pred.log.tips8.kg$plant.id)
shrub.count <- length(shrubs.id) ## how many shrubs

## vectors to hold results
lwr.shrub <- 1:shrub.count
upr.shrub <- 1:shrub.count
expcted.shrub <- 1:shrub.count

## loop over shrubs
for(i in 1:shrub.count){
```

```r
    temp.tips <- subset(pred.log.tips8.kg,plant.id==shrubs.id[i])
    temp.frustra <- subset(pred.frust8.kg,plant.id==shrubs.id[i])
## loop over simulations as one of every shrub and sum for the whole plot
## but do so n times, saving the total mass in kg
  for(j in 1:n){ ## sample each shrub n times
    ## sample the tips in each shrub
      how.many.tips <- length(temp.tips$fit)
      mean.tip.log.kg <- temp.tips$fit ## the expected value of tip biomass
      se.tip.log.kg <- sqrt(temp.tips$var.log) ## the standard error of tips
    ## sample each tip randomly from "lognormal" being sure to adjust lognormal mean
    tip.samples <- rlnorm(how.many.tips,
                   mean.tip.log.kg-(se.tip.log.kg^2)/2, ## important!
                   se.tip.log.kg)
    ## sum to get estimate of biomass for the shrub in this simulation
    rando.tips <- sum(tip.samples)
    ## sample the internodes in each plot
      how.many.frust <- length(temp.frustra$fit)
      mean.frust.kg <- temp.frustra$fit
      se.frust.kg <- sqrt(temp.frustra$var.kg)
    frust.samples <- rlnorm(how.many.frust,
                            mean.frust.kg-(se.frust.kg^2)/2,
                            se.frust.kg)
    ## sum them
    rando.frust <- sum(frust.samples)
    ## add up all the pieces for this plot simulation
    rando[j] <- (rando.tips + rando.frust)
  }
    ## Find the 95% quantile endpoints and expectation for each sample plot
 lwr.shrub[i] <- quantile(rando,p=0.025,na.rm=T)
 upr.shrub[i] <- quantile(rando,p=0.975,na.rm=T)
 expcted.shrub[i] <- mean(rando,na.rm=T)
}

## save summaries as dataframe
dat8.est <- data.frame(plant.id=shrubs.id,
                  shrub.est.kg=round(expcted.shrub,1),
                  lwr.kg=round(lwr.shrub,1),
                  upr.kg=round(upr.shrub,1),
                  unc.kg=round(I(upr.shrub-lwr.shrub),1))
## display
dat8.est <- dat8.est[order(dat8.est$shrub.est.kg),]
rownames(dat8.est) <- 1:dim(dat8.est)[1]
dat8.est
```

```
##   plant.id shrub.est.kg lwr.kg upr.kg unc.kg
## 1    APU2a          1.4    0.6    2.7    2.1
## 2    APU2d          2.0    0.9    4.0    3.1
## 3    APU2b          3.5    2.2    5.5    3.3
## 4    APU2c          4.0    2.6    6.0    3.4
## 5     APU1         14.4   11.1   18.6    7.5
## 6      JB1         40.9   33.4   50.4     17
## 7     APU3         65.4   56.5   75.7   19.2
## 8    JBER2        372.3  336.8  411.9   75.1
```

Comparing shrub estimates

Now let's compare the two-component estimate to the single-component estimate for each shrub:

```
compare.shrub <- merge(dat8.est,drc.dat8.est,by="plant.id")
compare.shrub.est <- merge(compare.shrub,wet.mass8.kg,by="plant.id")
RMSE.2comp <- with(compare.shrub.est,
                   sqrt(mean((shrub.est.kg-wet.mass.kg)^2)))
RMSE.1comp <- with(compare.shrub.est,
                   sqrt(mean((drc.est.kg-wet.mass.kg)^2)))
names(compare.shrub.est)
```

```
##  [1] "plant.id"     "shrub.est.kg" "lwr.kg"       "upr.kg"       "unc.kg"
##  [6] "drc.est.kg"   "drc.lwr.kg"   "drc.upr.kg"   "drc.unc.kg"   "wet.mass.kg"
## [11] "diam.cm"      "pieces.count"
```

Below we see clearly that for shrubs with DRC > 10.2 cm (shown in red), the two component model does much better in terms of precision—as indicated by the horizontal 95% prediction intervals—and in accuracy—as suggested by the closeness of the vertical red bars as point estimates very near the observed = predicted line.

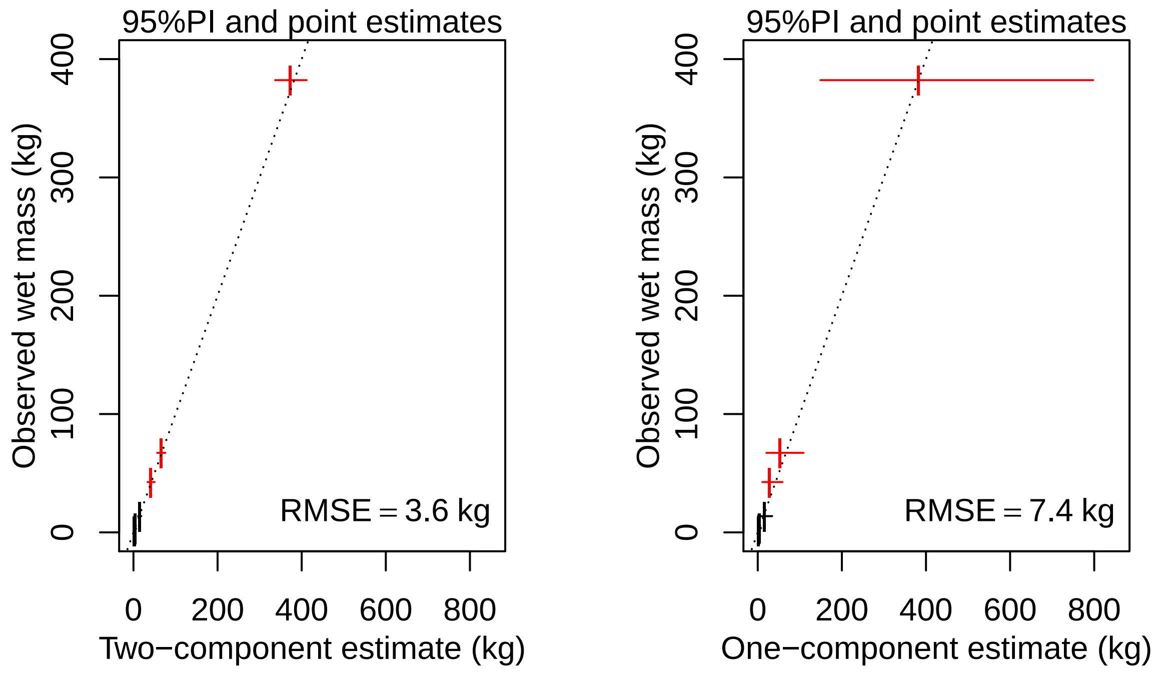

Using a t-test on the logged uncertainties suggest that the eidth of 95%PI was almost 2.5x larger on average using single-component allometrty than two-componment.

```
t.test(log(compare.shrub.est$drc.unc.kg),log(compare.shrub.est$unc.kg),
       paired=T,alternative="greater")
```

```
##
##  Paired t-test
##
## data:  log(compare.shrub.est$drc.unc.kg) and log(compare.shrub.est$unc.kg)
## t = 3.3463, df = 7, p-value = 0.006156
## alternative hypothesis: true difference in means is greater than 0
## 95 percent confidence interval:
##  0.3870562       Inf
## sample estimates:
```

```
## mean of the differences
##               0.8921717
```

```r
exp(0.9025945)
```

```
## [1] 2.465993
```

No significant difference in point estimates, but this would change if only estimates for shrubs with DRC > 10.2 cm were compared, when it seems likely that the two-component method would give perhaps slightly larger values than the one-component.

```r
t.test(log(compare.shrub.est$shrub.est.kg),log(compare.shrub.est$drc.est.kg),
       paired=T)
```

```
##
##  Paired t-test
##
## data:  log(compare.shrub.est$shrub.est.kg) and log(compare.shrub.est$drc.est.kg)
## t = 1.7055, df = 7, p-value = 0.1319
## alternative hypothesis: true difference in means is not equal to 0
## 95 percent confidence interval:
##  -0.04374738  0.27013959
## sample estimates:
## mean of the differences
##               0.1131961
```

```r
exp(0.1090787 )
```

```
## [1] 1.11525
```

Simulating Plot-level Shrub Biomass Distributions with Monte Carlo Sampling

Next we'll use a dataset of shrubs measured in southcentral Alaska in 2019.

```r
all.shrubs <- read.csv("../Data/The2019shrubs.csv")
str(all.shrubs)
```

```
## 'data.frame':    2019 obs. of  5 variables:
##  $ diam.cm  : num  0.94 1.65 1.78 2.01 2.03 ...
##  $ diam2.cm : num  0 0 0 0 0 0 0 0 0 0 ...
##  $ length.cm: num  0 0 0 0 0 0 0 0 0 0 ...
##  $ Plot     : Factor w/ 17 levels "CC1","HF1","HF2",..: 3 10 10 5 6 10 10 10 3 10 ...
##  $ plant.id : Factor w/ 1430 levels "CC1.1","CC1.10",..: 120 859 914 243 377 931 855 865 129 940 ...
```

Error Propagation with Single-component Allometry at Sample-plot Scale

First we'll use the single-measure allometry model to estimate biomass by plot, propagating the errors of each shrub through to the sum. The algorithm uses Monte Carlo sampling of lognormal distributions for each individual shrub, summing these, and then finding the middle 95%CI of biomass estimates.

1. Find the expected mean and se of each shrub's biomass recognizing that the field-mass estimate is lognormally distributed.
2. For each shrub, sample from its lognormally distributed mass estimate 10,000 times with mean given by the expected log of field-mass and prediction uncertainty given by $\texttt{residual.scale}^2 + \texttt{se.fit}^2$.
3. For each sample-plot of 10,000 simulations, sum the shrub masses, find the mean, and the 0.025 and 0.975 quantile to give the point estimate and an estimate of propagated error.

First, find the DRC for each individual shrub:

```
all.shrubs.DRC <- aggregate(diam.cm~plant.id+Plot,
  FUN=max,data=all.shrubs)
str(all.shrubs.DRC)
```

```
## 'data.frame':    1430 obs. of  3 variables:
##  $ plant.id: Factor w/ 1430 levels "CC1.1","CC1.10",..: 1 2 3 4 5 6 7 8 9 10 ...
##  $ Plot    : Factor w/ 17 levels "CC1","HF1","HF2",..: 1 1 1 1 1 1 1 1 1 1 ...
##  $ diam.cm : num  9.27 10.41 4.55 17.53 11.18 ...
```

```
range(all.shrubs.DRC$diam.cm)
```

```
## [1]  2.5400 30.3784
```

Then apply the single-measure DRC allometry:

```
pred.log.shrubs.kg <- predict(mod,
  newdata=all.shrubs.DRC,
  se=T) %>% data.frame()

pred.log.shrubs.kg$var.log <- pred.log.shrubs.kg$se.fit^2 + pred.log.shrubs.kg$residual.scale^2

pred.log.shrubs.kg$Plot <- all.shrubs.DRC$Plot
tail(pred.log.shrubs.kg)
```

```
##              fit     se.fit  df residual.scale   var.log Plot
## 1425 2.311337193 0.03914356 132      0.4275984 0.1843726  PH6
## 1426 1.214994083 0.05533636 132      0.4275984 0.1859025  PH6
## 1427 0.002147801 0.08132812 132      0.4275984 0.1894547  PH6
## 1428 0.422150955 0.07185442 132      0.4275984 0.1880035  PH6
## 1429 1.531230220 0.04956824 132      0.4275984 0.1852974  PH6
## 1430 2.633614624 0.03723001 132      0.4275984 0.1842265  PH6
```

Now apply Monte Carlo sampling to simulate field-mass sums and find quantiles for the distributions.

```
## n random simulations
n <- 10000
rando.shrubs <- 1:n ## one field-sample plot simulation

## plots
plots <- levels(pred.log.shrubs.kg$Plot)
plot.count <- length(plots) ## how many sample plots

## vectors to hold results
lwr <- 1:plot.count
upr <- 1:plot.count
mean.plot <- 1:plot.count

## loop over sample plots
for(i in 1:plot.count){
    temp.shrubs <- subset(pred.log.shrubs.kg,Plot==plots[i])

## loop over simulations as one of every shrub and sum for the whole plot
## but do so n times, saving the total mass in kg
  for(j in 1:n){ ## in each plot sample n times
    ## sample the tips in each plot
      how.many.shrubs <- length(temp.shrubs$fit)
```

```
      mean.shrubs.log.kg <- temp.shrubs$fit ## the expected value of tip biomass
      se.shrubs.log.kg <- sqrt(temp.shrubs$var.log) ## the standard errr of tips
    ## sample each tip randomly from "lognormal" being sure to adjust lognormal mean
    shrub.samples <- rlnorm(how.many.shrubs,
                    mean.shrubs.log.kg-(se.shrubs.log.kg^2)/2, ## important!
                    se.shrubs.log.kg)
    ## sum to get estimate of biomass for the plot in this simulation
    rando.shrubs[j] <- sum(shrub.samples)
  }
    ## Find the 95% quantile endpoints and expectation for each sample plot
 lwr[i] <- quantile(rando.shrubs,p=0.025,na.rm=T)
 upr[i] <- quantile(rando.shrubs,p=0.975,na.rm=T)
 mean.plot[i] <- mean(rando.shrubs,na.rm=T)
}

## save summaries as dataframe
dat.DRC <- data.frame(Plot=plots,
                  drc.plot.est.kg=round(mean.plot,1),
                  drc.lwr.kg=round(lwr,1),
                  drc.upr.kg=round(upr,1),
                  drc.range.kg=round(I(upr-lwr)))
## display
dat.DRC <- dat.DRC[order(dat.DRC$Plot),]
rownames(dat.DRC) <- 1:dim(dat.DRC)[1]
dat.DRC
```

```
##    Plot drc.plot.est.kg drc.lwr.kg drc.upr.kg drc.range.kg
## 1   CC1          1238.4     1058.3     1460.9          403
## 2   HF1           147.7      121.9      180.0           58
## 3   HF2           263.8      216.5      324.0          107
## 4   HF3           517.3      455.5      593.9          138
## 5   HF4           356.2      307.6      412.8          105
## 6   HF5           309.3      269.1      357.3           88
## 7   HP1          1291.0      987.0     1709.4          722
## 8   HP2           828.3      774.1      885.1          111
## 9   HP3           395.1      356.7      439.1           82
## 10  HP4          1686.5     1542.1     1846.2          304
## 11  HP5           494.2      449.4      544.7           95
## 12  PH1           235.7      181.2      322.5          141
## 13  PH2           559.1      475.7      659.7          184
## 14  PH3           391.4      324.3      471.6          147
## 15  PH4           386.4      318.2      467.6          149
## 16  PH5           490.0      416.6      577.8          161
## 17  PH6           621.5      552.2      704.4          152
```

Error Propagation with Two-component Allometry at Sample-plot Scale

Here we simulate the lognormal distribution of each shrub piece using the two-component allometry of frustra and tips following these steps:

1. Find the expected mean and se of each shrub piece's biomass recognizing that the field-mass of tip and internode estimates are lognormally distributed.
2. For tips and internodes sample from lognormal distributions 10,000 times with mean given by the expected log of field-mass and prediction uncertainty given by $\texttt{residual.scale}^2 + \texttt{se.fit}^2$.

3. For each sample-plot of 10,000 simulations, sum the shrub pieces, find the mean, and the 0.025 and 0.975 quantile to give the point estimate and an estimate of propagated error.

First we calculate internode volumes:

```
## internode volumes

all.shrubs$volume.cm3 <- (pi/3)*all.shrubs$length.cm*((all.shrubs$diam.cm/2)^2 +
       (all.shrubs$diam2.cm/2)^2 +
       (all.shrubs$diam2.cm/2)*(all.shrubs$diam.cm/2))
all.shrubs$volume.liter <- all.shrubs$volume.cm3/1000

all.shrubs <- all.shrubs[,c("Plot","volume.liter","diam.cm")]
```

We need to treat tips and internodes separately.

```
all.shrubs.tips <- subset(all.shrubs,volume.liter == 0 )
all.shrubs.frust <- subset(all.shrubs,volume.liter != 0 )
```

Make predictions for tips:

```
pred.log.tips.kg <- predict(all.under7.5,
  newdata=all.shrubs.tips,
  se=T) %>% data.frame()

pred.log.tips.kg$var.log <- pred.log.tips.kg$se.fit^2 + pred.log.tips.kg$residual.scale^2

pred.log.tips.kg$Plot <- all.shrubs.tips$Plot
```

Make predictions for internodes:

```
pred.frust.kg <- predict(frustrum.mod,
  newdata=all.shrubs.frust,
  se=T) %>% data.frame()

pred.frust.kg$var.kg <- pred.frust.kg$se.fit^2 + pred.frust.kg$residual.scale^2

pred.frust.kg$Plot <- all.shrubs.frust$Plot
```

Monte Carlo simulation of field-mass sums and find quantiles for the lognormal distributions of shrubs in plots using two-component model.

```
## n random simulations
n <- 10000
rando <- 1:n ## one field-sample plot simulation

## plots
plots <- levels(pred.log.tips.kg$Plot)
plot.count <- length(plots) ## how many sample plots

## vectors to hold results
lwr <- 1:plot.count
upr <- 1:plot.count
mean.plot <- 1:plot.count

## loop over sample plots
for(i in 1:plot.count){
```

```
    temp.tips <- subset(pred.log.tips.kg,Plot==plots[i])
    temp.frustra <- subset(pred.frust.kg,Plot==plots[i])
## loop over simulations as one of every shrub and sum for the whole plot
## but do so n times, saving the total mass in kg
  for(j in 1:n){ ## in each plot sample n times
    ## sample the tips in each plot
      how.many.tips <- length(temp.tips$fit)
      mean.tip.log.kg <- temp.tips$fit ## the expected value of tip biomass
      se.tip.log.kg <- sqrt(temp.tips$var.log) ## the standard errr of tips
    ## sample each tip randomly from "lognormal" being sure to adjust lognormal mean
    tip.samples <- rlnorm(how.many.tips,
                    mean.tip.log.kg-(se.tip.log.kg^2)/2, ## important!
                    se.tip.log.kg)
    ## sum to get estimate of biomass for the plot in this simulation
    rando.tips <- sum(tip.samples)
    ## sample the internodes in each plot
      how.many.frust <- length(temp.frustra$fit)
      mean.frust.kg <- temp.frustra$fit
      se.frust.kg <- sqrt(temp.frustra$var.kg)
    frust.samples <- rlnorm(how.many.frust,
                            mean.frust.kg-(se.frust.kg^2)/2,
                            se.frust.kg)
    ## sum them
    rando.frust <- sum(frust.samples)
    ## add up all the pieces for this plot simulation
    rando[j] <- (rando.tips + rando.frust)
  }
    ## Find the 95% quantile endpoints and expectation for each sample plot
 lwr[i] <- quantile(rando,p=0.025,na.rm=T)
 upr[i] <- quantile(rando,p=0.975,na.rm=T)
 mean.plot[i] <- mean(rando,na.rm=T)
}

## save summaries as dataframe
dat <- data.frame(Plot=plots,
                  plot.est.kg=round(mean.plot,1),
                  lwr.kg=round(lwr,1),
                  upr.kg=round(upr,1),
                  range.kg=round(I(upr-lwr)))
## display
dat <- dat[order(dat$Plot),]
rownames(dat) <- 1:dim(dat)[1]
dat
```

```
##    Plot plot.est.kg lwr.kg upr.kg range.kg
## 1   CC1      2257.9 2145.6 2377.0      231
## 2   HF1       162.0  136.7  192.1       55
## 3   HF2       242.8  214.6  275.9       61
## 4   HF3       498.8  456.1  545.6       89
## 5   HF4       377.2  335.4  424.9       89
## 6   HF5       307.8  272.0  348.3       76
## 7   HP1      1409.1 1320.8 1503.5      183
## 8   HP2       878.4  828.3  931.8      104
## 9   HP3       422.1  387.8  461.0       73
```

```
## 10  HP4       1863.1 1752.7 1980.8      228
## 11  HP5        524.8  483.3  570.3       87
## 12  PH1        249.2  194.7  330.4      136
## 13  PH2        665.9  610.2  728.3      118
## 14  PH3        434.7  386.5  489.8      103
## 15  PH4        353.6  318.4  393.3       75
## 16  PH5        529.2  475.2  591.3      116
## 17  PH6        651.4  599.3  709.4      110
```

Comparing the sample-plot estimates found by the two-component model to the single-component model

```
(merged <- merge(dat[,c("Plot","plot.est.kg","upr.kg","lwr.kg","range.kg")],
  dat.DRC,by="Plot"))
```

```
##    Plot plot.est.kg upr.kg lwr.kg range.kg drc.plot.est.kg drc.lwr.kg
## 1   CC1      2257.9 2377.0 2145.6      231          1238.4     1058.3
## 2   HF1       162.0  192.1  136.7       55           147.7      121.9
## 3   HF2       242.8  275.9  214.6       61           263.8      216.5
## 4   HF3       498.8  545.6  456.1       89           517.3      455.5
## 5   HF4       377.2  424.9  335.4       89           356.2      307.6
## 6   HF5       307.8  348.3  272.0       76           309.3      269.1
## 7   HP1      1409.1 1503.5 1320.8      183          1291.0      987.0
## 8   HP2       878.4  931.8  828.3      104           828.3      774.1
## 9   HP3       422.1  461.0  387.8       73           395.1      356.7
## 10  HP4      1863.1 1980.8 1752.7      228          1686.5     1542.1
## 11  HP5       524.8  570.3  483.3       87           494.2      449.4
## 12  PH1       249.2  330.4  194.7      136           235.7      181.2
## 13  PH2       665.9  728.3  610.2      118           559.1      475.7
## 14  PH3       434.7  489.8  386.5      103           391.4      324.3
## 15  PH4       353.6  393.3  318.4       75           386.4      318.2
## 16  PH5       529.2  591.3  475.2      116           490.0      416.6
## 17  PH6       651.4  709.4  599.3      110           621.5      552.2
##    drc.upr.kg drc.range.kg
## 1      1460.9          403
## 2       180.0           58
## 3       324.0          107
## 4       593.9          138
## 5       412.8          105
## 6       357.3           88
## 7      1709.4          722
## 8       885.1          111
## 9       439.1           82
## 10     1846.2          304
## 11      544.7           95
## 12      322.5          141
## 13      659.7          184
## 14      471.6          147
## 15      467.6          149
## 16      577.8          161
## 17      704.4          152
```

We see that the paired samples t-test suggests a difference between the two methods for estimating total sample-plot biomass. It would seem that the two-component method is more accurate as it is based on pieces

of shrubs.

```
with(merged,
     t.test(log(drc.plot.est.kg),
       log(plot.est.kg),paired=T))
```

```
##
##  Paired t-test
##
## data:  log(drc.plot.est.kg) and log(plot.est.kg)
## t = -2.2124, df = 16, p-value = 0.04183
## alternative hypothesis: true difference in means is not equal to 0
## 95 percent confidence interval:
##  -0.157667195 -0.003365957
## sample estimates:
## mean of the differences
##             -0.08051658
```

In addition, the two-component method is more precise, since the difference in the log of 95%CI range (i.e., uncertainty) is significant.

```
ttest <- with(merged,
     t.test(log(drc.range.kg),log(range.kg),
            paired=T,
            alternative="greater"))
ttest
```

```
##
##  Paired t-test
##
## data:  log(drc.range.kg) and log(range.kg)
## t = 4.4386, df = 16, p-value = 0.0002064
## alternative hypothesis: true difference in means is greater than 0
## 95 percent confidence interval:
##  0.2150216       Inf
## sample estimates:
## mean of the differences
##               0.3544382
```

Moreover, since the mean difference in the log(uncertainty) is 0.3544382, the mean ratio of the uncertainties is 1.43, suggesting that the two-component method is about 40% more precice.

```
write.csv(merged,file="../Data/SI_LogNormal_Sim.csv",
  row.names=F)
```
